# Patient and Regimen Characteristics Associated with Self-Reported Nonadherence to Antiretroviral Therapy

**DOI:** 10.1371/journal.pone.0000552

**Published:** 2007-06-20

**Authors:** Patrick S. Sullivan, Michael L. Campsmith, Glenn V. Nakamura, Elin B. Begley, Jeffrey Schulden, Allyn K. Nakashima

**Affiliations:** Centers for Disease Control and Prevention, Atlanta, Georgia, United States of America; AIDS Research Center, Chinese Academy of Medical Sciences and Peking Union Medical College, China

## Abstract

**Background:**

Nonadherence to antiretroviral therapy (ARVT) is an important behavioral determinant of the success of ARVT. Nonadherence may lead to virological failure, and increases the risk of development of drug resistance. Understanding the prevalence of nonadherence and associated factors is important to inform secondary HIV prevention efforts.

**Methodology/Principal Findings:**

We used data from a cross-sectional interview study of persons with HIV conducted in 18 U.S. states from 2000–2004. We calculated the proportion of nonadherent respondents (took <95% of prescribed doses in the past 48 hours), and the proportion of doses missed. We used multivariate logistic regression to describe factors associated with nonadherence. Nine hundred and fifty-eight (16%) of 5,887 respondents reported nonadherence. Nonadherence was significantly (p<0.05) associated with black race and Hispanic ethnicity; age <40 years; alcohol or crack use in the prior 12 months; being prescribed ≥4 medications; living in a shelter or on the street; and feeling “blue” ≥14 of the past 30 days. We found weaker associations with having both male-male sex and injection drug use risks for HIV acquisition; being prescribed ARVT for ≥21 months; and being prescribed a protease inhibitor (PI)-based regimen not boosted with ritonavir. The median proportion of doses missed was 50%. The most common reasons for missing doses were forgetting and side effects.

**Conclusions/Significance:**

Self-reported recent nonadherence was high in our study. Our data support increased emphasis on adherence in clinical settings, and additional research on how providers and patients can overcome barriers to adherence.

## Introduction

The full benefit of antiretroviral therapy (ARVT) in HIV-infected patients requires patients to be prescribed appropriate therapy, the virus to be sensitive to the prescribed therapy, and patients to adhere to the prescribed regimen [1]. Evidence from clinical trials suggests that very high levels of adherence (>95%) are required for optimally suppressing viral load and preventing the emergence of resistant virus [2], [3]. Even among those with low CD4 counts at the initiation of ARVT, adherence is a strong predictor of increase in CD4 count after initiation of combination ARVT [4]. There may also be significant public health benefits associated with prescription of antiretroviral therapy and good adherence: on a population basis, lower viral loads are predicted to be associated with decreased sexual [5], perinatal [6], and injection-related [7] transmission of HIV.

Therefore, understanding the prevalence of nonadherence, its associated factors, and reasons for nonadherence is an important clinical and public health goal. Behaviors, such as adherence, that are related to HIV disease progression are measured as part of behavioral surveillance projects conducted in the United States [8]. Generally, behavioral surveillance systems measure behaviors of interest in a large number of persons, but do not collect the same depth of information on specific topics that might be collected in smaller research studies.

Although many factors have been consistently reported to be associated with poor adherence (reviewed by Reynolds [9] and Ammasarri [10]), other associations are less clear. For many key demographic factors, as well as certain social and regimen-related factors, contradictory results appear in published literature [10]. Most recently, there have been contradictory reports as to whether some types of ARVT regimens (e.g., non-nucleoside reverse transcriptase inhibitor [NNRTI] -based regimens) may be associated with higher levels of adherence [11]–[16].

Methods for studying adherence are varied. They include electronic monitoring of access to medication bottles, examination of pharmacy records, physician estimation, and measurement of drug levels [17]–[21]. These methods are costly, however, and may be difficult to implement in large behavioral surveillance projects. Although self-reported adherence is subject to limitations common to interview studies and likely overestimates adherence [18], self-report provides a measure of adherence which generally correlates well with more objective methods [21]–[26] and with virologic treatment response [27]. It is also practical for use in ongoing behavioral surveillance efforts. We report the results of an exploratory analysis of behavioral surveillance data on self-reported adherence from a multi-state supplemental surveillance study of persons with HIV infection.

## Methods

### Project

The Supplement to HIV and AIDS Surveillance (SHAS) Project is a cross-sectional behavioral surveillance study of persons with HIV infection. The methods have been previously described [28]. Briefly, adults, aged 18 years and older, reported with HIV or AIDS through routine case surveillance were eligible for participation. Participants were enrolled using one of two methods: 1) facility-based recruitment of all eligible persons seeking treatment at selected health care facilities in Denver, Colorado; Hartford and New Haven, Connecticut; Jacksonville, Miami, and Tampa, Florida; Atlanta, Georgia; Chicago, Illinois; Baltimore, Maryland; Detroit, Michigan; Jersey City and Paterson, New Jersey; and Philadelphia, Pennsylvania; or 2) population-based recruitment of all eligible persons in Phoenix and Tucson, Arizona; Delaware; Kansas; Los Angeles County, California; Minneapolis/St. Paul, Minnesota; New Mexico; South Carolina; Austin and Houston, Texas; and Washington. All sites interviewed persons reported with AIDS. Additionally, during the time period examined for this analysis, Arizona, Denver, Detroit, Florida, Kansas, Minnesota, New Jersey, New Mexico, South Carolina and Texas also interviewed persons with HIV infection which had not progressed to AIDS. Informed consent was obtained from all participants prior to the interview, and the study received institutional review board (IRB) approval at both the Centers for Disease Control and Prevention and local levels.

### Measures

Beginning in May 2000, the SHAS Project added questions on medication history, adherence, and reasons for nonadherence. We utilized data from interviews conducted from May 2000 through June 2004 (when the project ended). Respondents who were prescribed antiretroviral therapy at the time of the interview were asked, “In the last 48 hours, have you missed or skipped any pills or spoonfuls of any of your antiretroviral medications?” We collected detailed information about the prescribed antiretroviral regimens at the time of the interview, including the number of daily doses prescribed for each medication. For respondents who reported missing one or more doses of medication in the 48 hours before interview, we asked about the number of doses missed, and calculated the total proportion of doses taken. We defined nonadherence as taking less than 95% of prescribed doses in the 48 hours before interview. We also asked the reasons why doses were missed and field coded the participants' responses using a list of possible reasons constructed by the investigators. Interviewers received training on administering the questionnaire, and used reference materials, including pictures of capsules of various medications, to help respondents identify their current prescriptions.

We defined protease inhibitor (PI)-based regimens as consisting of any 2 or more nucleoside/nucleotide reverse transcriptase inhibitors (NRTIs) plus any PI; boosted PI-based regimens as additionally including ritonavir; unboosted PI-based regimens as not including ritonavir; NNRTI-based regimens as consisting of any 2 or more NRTIs plus any NNRTI; triple NRTI therapy as consisting of any 3 or more NRTIs; and other ARVT as consisting of any one or more PI, NNRTI, NRTI, or pentafuside, but not meeting one of the previous definitions.

### Analyses of All SHAS Respondents

We constructed a multivariate logistic regression model (PROC LOGISTIC, SAS Institute, Cary, NC) to calculate adjusted odd ratios and 95% confidence intervals for factors associated with nonadherence. We selected putative explanatory variables based on review of published biomedical literature. Our putative explanatory variables fell into several categories: demographic factors (age, race, sex, risk for acquiring HIV infection), socioeconomic status indicators (living situation, income, education, being on public assistance, and currently having health insurance) recent substance use behaviors (use of alcohol, crack, or injection drugs in the 12 months before the interview, and the CAGE questions [29] for possible alcohol abuse), characteristics of the ARVT regimen (type of regimen, number of drugs in the regimen, and duration of ARVT treatment), aspects of HIV care (use of a reminder system for medications, and whether the healthcare provider had discussed antiretroviral resistance with the patient), and indicators of depression (“feeling blue” for ≥14 of the past 30 days, having limited activity for ≥14 of the past 30 days, reporting a current met or unmet need for mental health services, and history of incarceration).

To construct our model, we first examined correlation coefficients of all pairs of putative explanatory variables. Where significant correlations existed, we removed one of the two correlated variables from consideration. For example, being on public assistance was removed from consideration, but current income was retained for consideration. We conducted bivariate analyses for all remaining explanatory variables, and only those with a p value of <0.25 from chi-square testing were considered in subsequent model building. Next we built a multivariate logistic regression model using manual forward selection. At each step, the remaining variable with the largest chi-square for its association with nonadherence was entered into the model, and inspected to determine if the associated p value in the model was <0.05. Factors continued to be entered until the last factor entered had a p value of >0.05. Once factors were entered into the model, they were not removed. The model was complete with respect to first order terms when no other variable could be entered and retained with a p value of <0.05. After all first order terms were entered into the model, we checked all two-way interaction terms, using an experiment-wide alpha of 0.05 because of the large number of two-way terms tested. No significant two-way interaction term was identified.

We conducted a similar process of modeling, in which, for certain factors with multiple levels – race, age, risk for HIV acquisition, ARVT regimen type, regimen duration, and number of drugs in regimen – if any level of the factor was significant, all levels of the factor would be retained in the model, regardless of significance. The results of this model were essentially the same as the reduced model, so we chose to present the reduced model.

For those who reported nonadherence, we also calculated the proportion of doses missed in the 48 hours before the interview, and tabulated the main reasons reported for missing doses.

### Subgroup Analysis: Validation of Self-Reported Adherence Using Viral Load Measurements

We validated our self-reported adherence outcome measure by using data from a subset of SHAS respondents prescribed ARVT at the time of interview who were also observed in the Adult and Adolescent Spectrum of HIV Disease (ASD) Project (a longitudinal medical records review project conducted in some of the same project areas as SHAS) [30]. For those who were in both SHAS and ASD datasets, we determined the lowest HIV RNA concentration during the 6 month period including the SHAS interview date for each respondent. The distributions of HIV RNA concentration were not normal for either those who reported nonadherence or those who did not report nonadherence. Therefore, the median HIV RNA concentrations between the two groups were compared using the Wilcoxon Rank Sum test.

## Results

From May 2000 through June 2004, 11,503 persons were offered enrollment in SHAS; 9,088 (79%) were interviewed, and 2,415 (21%) refused to participate. Thirty-nine of 5,926 respondents who were prescribed ARVT at the time of interview had insufficient information on daily doses prescribed or number of doses missed in the 48 hours before the interview, and were, therefore, excluded. Thus, for this analysis, we included 5,887 (65% of interviewed) respondents who had complete information on antiretroviral prescription and dosing and who reported being prescribed at least one antiretroviral medication at the time of interview. Out of 5,887 respondents, 520 had been interviewed in a state where ASD was also conducted, and had data from medical records abstractions that spanned the date of the SHAS interview. The 520 were included in the subgroup analyses for validation of self-reported adherence using viral loads. The characteristics of interviewed persons, persons who were prescribed ARVT at the time of interview and included in the analysis of nonadherence, and persons included in the subgroup viral load analysis, are reported in Table 1.

**Table 1:** Characteristics of persons with HIV infection interviewed in the Supplement to HIV and AIDS Surveillance Project, who were prescribed antiretroviral drugs at the time of interview, and who were included in subgroup analyses of viral load, 18 US states, 2000–2004

Characteristic	All Respondents* N (%)	Prescribed antiretroviral drugs at time of interview n (%)	Included in subgroup analysis of viral load n (%)
Sex			
Male	6421 (72)	4333 (74)	337 (73)
Female	2487 (28)	1554 (26)	124 (27)
Race			
White, non-Hispanic	1983 (22)	1393 (24)	138 (30)
Black, non-Hispanic	4883 (55)	3014 (51)	221 (48)
Hispanic	1709 (19)	1262 (21)	88 (19)
Other†	333 (4)	218 (4)	14 (3)
Age at interview (years)			
18–29	1144 (13)	633 (11)	60 (13)
30–39	3232 (36)	2113 (36)	157 (34)
> = 40	4532 (51)	3141 (53)	244 (53)
Risk for HIV infection			
Male-male sex	3540 (40)	2480 (42)	220 (48)
Injection drug use	1377 (15)	888 (15)	66 (14)
Male-male sex and injection drug use	724 (8)	466 (8)	46 (10)
Male-female sex	1788 (20)	1107 (19)	60 (13)
Other risks‡	1479 (17)	946 (16)	69 (15)
AIDS Diagnosis			
AIDS	5545 (62)	4336 (74)	337 (73)
HIV infection, no AIDS	3241 (36)	1551 (26)	124 (27)
Undetermined	122 (1)	0 (0)	0 (0)

* Excludes 180 respondents with incomplete responses on prescription of antiretroviral therapy

† Other races include non-Hispanic American Indian/Alaskan Native, non-Hispanic Asian/Pacific Islander, and persons reporting multiple races.

‡ Other risks include blood or blood product transfusion and unknown risks.

### Analyses of All SHAS Respondents

Among all SHAS respondents prescribed at least one antiretroviral medication at the time of the interview, 958 (16%) reported nonadherence in the 48 hours before the interview. The results of multivariate logistic regression (Table 2) indicated that several groups were significantly (p<0.05) more likely to report nonadherence: black non-Hispanic and Hispanic respondents (versus all other races); respondents aged 18–29 or 30–39 years (versus those aged ≥40 years); respondents who reported using alcohol or crack cocaine in the past 12 months; respondents prescribed ≥4 medications at the time of interview (versus 1–3 medications); respondents who had been blue for ≥14 of the past 30 days; and respondents currently living in a shelter or on the street (versus all other living situations). Respondents living in a medical facility were less likely to report nonadherence. Several additional factors were significantly associated with nonadherence, but had a weak or modest strength of association and lower bounds of the 95% CI close to 1.0. Such weak associations were observed for having both male-male sex and injecting drug use risks for HIV acquisition (versus all other acquisition modes); being prescribed an unboosted PI-based regimen (versus any other regimen); and being prescribed ARVT for ≥21 months (versus <21 months).

**Table 2:** Characteristics of HIV infected persons interviewed about adherence to currently prescribed antiretroviral therapy, number and proportion of nonadherent respondents, and logistic regression model of factors associated with nonadherence among 5,887 respondents to the Supplement to HIV/AIDS Surveillance Project, 18 US States, 2000 to 2004

Characteristic	No. interviewed	No. of respondents <95% adherence in prior 48 hours (%)	Adjusted Odds Ratio (95% CI)
*Demographics*			
Race			
Black, non-Hispanic*	3014	584 (19)	2.0 (1.6–2.4)
Hispanic*	1262	183 (15)	1.4 (1.1–1.8)
Age (years)			
18–29†	633	121 (19)	1.4 (1.2–1.8)
30–39†	2113	361 (17)	1.3 (1.0–1.4) ‡
Risk for HIV infection is male-male sex and injection drug use§	466	87 (19)	1.3 (1.0–1.7) ‡
Place of residence			
Medical facility∥	117	10 (9)	0.4 (0.2–0.7)
Shelter or street∥	156	47 (30)	1.8 (1.2–2.6)
*Substance use in past year*			
Alcohol use in past year¶	3573	630 (18)	1.3 (1.1–1.5)
Crack use in past year¶	624	165 (26)	1.7 (1.4–2.1)
*Characteristics of ARVT regimen*			
Duration of ARVT treatment ≥21 months**	3023	521 (17)	1.2 (1.0–1.3) ‡
Currently prescribed ≥4 ARVT medications††	704	160 (23)	1.6 (1.3–2.0)
Currently prescribed unboosted PI-based HAART‡‡	1524	282 (19)	1.2 (1.0–1.4) ‡
*Mental Health Indicators*			
Felt blue 14 or more of last 30 days	172	37 (22)	1.6 (1.4–1.8)

ARVT: antiretroviral therapy; PI: protease inhibitor; HAART: Highly active antiretroviral therapy.

* Referent group is all other races

† Referent group is ≥40 years

‡ Confidence interval excludes 1.0; lower bound is rounded down to 1.0.

§ Referent group is all other risks

∥ Referent group is all other places of residence

¶ Any use in the 12 months before the interview

** Referent group is duration of ARVT treatment <21 months

†† Referent group is currently prescribed 1–3 drugs

‡‡ Referent group is any other prescribed regimen

Among the 958 persons who were nonadherent, the median proportion of doses missed was 50% of doses. The mode of missed doses was 100%; 226 of 958 (24%) of nonadherent respondents reported taking no dose of medication during the previous 48 hours.

A total of 821 persons (86% of those who missed ≥5% of doses in the 48 hours before the interview) reported the most important reason that doses were missed. The most commonly reported reasons were forgetting to take the medicine (266 respondents, 32%); side effects from the medications (152 respondents, 16%); inability to get to the clinic or doctor for a prescription (106 respondents, 11%); and inability to fit medications into the respondent's schedule (84 respondents, 9%). Other reported main reasons included, getting depressed (28 respondents, 3%); not believing that the medications work (21 respondents, 2%); and being on the street, in jail, or in prison (7 respondents, 1%).

### Subgroup Analysis: Validation of Self-Reported Adherence Using Viral Load Measurements

Results from the subgroup who had HIV RNA concentrations available for analysis indicated that the HIV RNA concentration was higher for the 64 persons who reported taking <95% of prescribed doses in the 48 hours before the interview (median  =  400; range: undetectable – 425,000) than for the 397 persons who took ≥95% of prescribed doses (median  =  85; range: undetectable – 750,000; p  =  0.03 by 2-sided Wilcoxon Rank Sum Test). The HIV RNA measurements were taken a median of 36 days from the interview (interquartile range, 6–78 days).

## Discussion

In a large, diverse group of persons living with HIV infection, we observed a magnitude of nonadherence that poses a considerable threat to the individual and public health benefits of ARVT. About 1 in 6 persons interviewed in our project had missed at least 5% of their prescribed ARVT doses in the 48 hours before the interview. Although we used a self-reported measure, the self-reported measure of adherence was related to virologic outcome among a subgroup of our respondents, as indicated by viral load measurement – a relevant biological marker associated with adherence status [27], [31]. This suggests that self-report of recent adherence was correlated with prior adherence in our respondents.

Our findings provide additional data in some areas where inconsistent or null findings have been reported in the literature. For example, a summary analysis of published studies on adherence [10] reported that age and race had been inconsistently associated with adherence in previously published multivariate analyses. We report associations between adherence and both age and race; our data agree with another recent report of higher adherence among older persons living with HIV infection [32]. The same summary of published literature [10] identified several factors which had not generally been associated with adherence in the published literature. These included risk factor for HIV infection, length of time on therapy, and number of antiretroviral drugs. Our analysis showed statistically significant associations with each of these factors, although for risk factor and time on therapy, our associations were marginally significant, especially in light of our large sample size.

Many investigators have reported lower adherence among persons with current substance use [33]–[37]. In contrast, Martini et al. [38] found that heroin, cocaine, and alcohol users were not more likely to report nonadherence. In our data, alcohol use and crack use, but not recent injection drug use, were associated with nonadherence.

Depression is a factor which has also been consistently associated with nonadherence [2], [16], [37], [39]–[43]. Although we did not have data on clinical diagnoses of depression, we included putative explanatory variables which might be associated with depression. For example, days of “feeling blue”, self-identified need for mental health services, and days of limited activity were considered in our modeling. Despite consistent evidence of the association between adherence and depression in the literature and a significant association between nonadherence and higher number of days feeling blue in our model, only 3% of our nonadherent respondents identified getting depressed as a main reason for missing doses.

Among respondents in our project, prescription of an unboosted – but not a boosted – PI-based regimen, was weakly associated with nonadherence. Weiser et al. [13] reported no difference in adherence between patients prescribed NNRTI versus PI-based regimens. Other researchers have reported lower adherence among patients prescribed PI-based regimens [12], [14], [16], while Glass et al. reported more nonadherence among patients prescribed a boosted PI-based regimen [15]. If adherence is truly higher in patients prescribed NNRTI-based regimens, it may be explained by a lower pill burden [14], [33], [44]. However, our analysis partially controlled for lower pill burden by controlling for the number of antiretroviral medications prescribed. Alternatively, higher adherence for those prescribed NNRTI regimens could be explained by fewer intolerable side effects. In our data, side effects were a commonly reported reason for missing doses among those who were nonadherent. Further, self-perception of body changes such as peripheral fat loss or central fat gain are associated with lower adherence [45], and may be more common among those taking PI-based ARVT [46].

Even if our finding of a marginal association of nonadherence with prescription of an unboosted PI-based regimen were confirmed by others, the clinical significance of this finding would be unclear. Recent findings [47] suggest that viral suppression may be maintained with less than 95% adherence for patients on boosted PI-based regimens. If this finding is substantiated in other settings, then future analyses of adherence and clinical guidelines may consider having regimen-specific thresholds to define acceptable adherence [48].

Pill burdens for patients currently on ARVT are likely to be significantly lower now than were pill burdens at the time of our interviews [33], [44]. This is because of new formulations which include multiple drugs in a single pill or capsule, and regimens which require fewer prescribed doses per day.

Although the most commonly reported reason for missed ARVT doses was forgetting to take medications, the modal proportion of doses missed was 100%. The fact that nearly a quarter of non-adherent respondents took no medication during the 48 hour period suggests that, in many cases, doses are not missed sporadically. More prolonged periods of missed doses could represent “drug holiday” periods [49], periods in which patients ran out of medication, or periods when significant side effects or illness prevented taking any. Future work should attempt to characterize the reasons for these prolonged periods of missed doses.

Our study has some important limitations. As with any interview study, our data are subject to recall bias and social desirability bias [50]. We believe asking about adherence in the 48 hours before the interview should minimize recall bias. Social desirability bias should lead to under-report of poor adherence, which has been recently confirmed [18]; this suggests that our data represent minimum estimates of nonadherence. However, social desirability bias has been reported not to be a major source of error in collecting self-reported adherence data [51]. The persons recruited and interviewed in the SHAS project are not representative of all persons with HIV infection or AIDS in the project areas, or in the United States.

Further, there are important factors which are likely related to nonadherence which the SHAS interview did not collect, and therefore could not be included in our analysis. For example, we did not collect information on social support or isolation [52] (other than current living arrangements [39]), beliefs about prognosis or treatment efficacy [53], [54], perceptions of body changes [45], cognitive factors [55], [56], serostatus disclosure [57], or life stress [54], [58].

These results have some relevance for clinicians and public health officials. Based on our findings and existing treatment guidelines [59], providers should consider that certain groups of patients may be especially likely to benefit from support and guidance around the importance of adherence and strategies for improving adherence. Our data, and the data of others, support referral to housing resources for homeless patients and referral to appropriate treatment for alcohol abuse, crack cocaine use, and mental health problems, either before or concurrently with prescription of antiretroviral medications. Some other factors associated with nonadherence among our respondents, such as race/ethnicity and age, are likely markers for other barriers that lead to poor adherence. Additional focused research is needed to determine the underlying challenges to adherence for these patients, and to develop interventions and resources to help physicians and patients overcome these challenges.

Adherence to antiretroviral medications also has important public health implications. Poor adherence to ARVT may be associated with the development of drug-resistant strains of HIV. Also, to the extent that ARVT lowers prevalent viral loads on a population basis, there should be a desirable impact on reducing the risk of HIV transmission. Poor adherence also compromises this potential population-level benefit of ARVT. Thus, adherence is a relevant behavior for targeting with interventions, and for monitoring as a part of future behavioral surveillance projects [60]. Although large-scale behavioral surveillance efforts are not designed to provide detailed clinical information about adherence and its consequences, and are subject to some important limitations, such systems offer the opportunity to measure important variables related to HIV care, including adherence, in an ongoing and systematic way.
